# Managing multiple long-term conditions in people experiencing socioeconomic deprivation: a qualitative study of primary care perspectives

**DOI:** 10.3399/BJGP.2025.0361

**Published:** 2026-03-10

**Authors:** Eleanor Wyke, Elizabeth R Baxter, Carolyn A Chew-Graham, Abi Woodward, Kate Walters, Nathan Davies, Stephanie JC Taylor, Jamie Ross, Joanne Protheroe, Caroline Mitchell, Megan Armstrong

**Affiliations:** 1 Research Department of Primary Care and Population Health, University College London, London, UK; 2 School of Medicine, Keele University, Keele, UK; 3 Centre for Psychiatry & Mental Health, Wolfson Institute of Population Health, Queen Mary University London, London, UK; 4 Centre for Primary Care, Wolfson Institute of Population Health, Queen Mary University London, London, UK

**Keywords:** comorbidity, general practice, primary health care, self-management, socioeconomic factors

## Abstract

**Background:**

Multiple long-term conditions (MLTCs) are rising, especially among people experiencing socioeconomic deprivation, who develop MLTCs earlier and face barriers to self-management. Primary healthcare professionals (HCPs) are central to supporting self-management but face systemic challenges, resource inequalities, and emotional strain. Understanding their perspectives is vital to developing effective solutions.

**Aim:**

To explore how HCPs in general practices in deprived areas support people to self-manage MLTCs, and the barriers to, and facilitators of, providing best levels of care.

**Design and setting:**

This was a qualitative study with GPs, general practice nurses, advanced nurse practitioners, and allied health professionals working in socioeconomically deprived areas of two cities in England.

**Method:**

Online semi-structured interviews were conducted with 18 HCPs from 17 general practices. Transcripts were analysed inductively, underpinned by elements of reflexive thematic analysis and grounded-theory methodology. A socioecological framework was applied retrospectively to situate HCP experiences within wider socioecological discourses.

**Results:**

Four themes were identified: 1) individual factors influencing self-management, such as motivation and health literacy; 2) the role of social connections and community-based support; 3) enhancing primary care through continuity, longer appointments, and culturally sensitive, person-centred care; and 4) the impact of policy and inequitable funding on care provision. Key facilitators included accessible information, community signposting, and support navigating healthcare systems.

**Conclusion:**

HCPs adapt care to meet patient needs but face systemic barriers, including underfunding and limited resources. Strengthening trust, cultural competence, and continuity of care, alongside policy and funding reform, is essential for delivering effective self-management support.

## How this fits in

Previous studies have examined multiple long-term condition (MLTC) self-management in people experiencing socioeconomic deprivation from the perspectives of patients and carers. The current study provides the perspectives of healthcare professionals in primary care teams who support self-management in socioeconomically deprived areas. It also highlights some of the political, economic, and social drivers that have an impact on the self-management support that primary care teams can provide. In addition, these research findings uncover facilitators to supporting MLTC self-management in these areas, which are crucial to reduce the increase and cyclical relationship of socioeconomic inequality and accumulation of MLTCs.

## Introduction

Multiple long-term conditions (MLTCs), that is, two or more long-term conditions in an individual, is predicted to rise in the UK for those aged ≥65 years from 54% in 2015 to 68% in 2035.^1^ The prevalence of MLTCs is associated with an increased demand on healthcare services and social care costs.^2,3^ People experiencing socioeconomic deprivation are those living with unmet basic needs caused by a lack of social and economic resources such as income, employment, education, housing, and access to essential services. This population is almost twice as likely to develop MLTCs and develop these on average 10–15 years earlier than those from more affluent groups.^4^ A recent microsimulation study predicts that socioeconomic inequalities in experiencing MLTCs will widen considerably, especially in the working-age population, and that socioeconomic inequalities will gradually increase with accumulation of MLTCs.^5^

Supporting people to self-manage their conditions is associated with improved outcomes for a variety of health conditions (for example, diabetes, asthma).^6^ Self-management involves:

- medical management;
- changing unhealthy or maintaining healthy behaviours; and
- emotional management.^7^

A qualitative systematic review exploring self-management of MLTCs in people experiencing socioeconomic deprivation found that while maintaining independence and engaging in meaningful activities to self-manage was important, because of their finances, health literacy levels, and lack of support networks, individuals seldom proactively self-managed their MLTCs.^8^ A recent ethnographic study explored how working-age participants living with MLTCs experience ‘chronic crisis’, which was viewed as an extension of the socially challenging conditions they have faced throughout their lives.^9^ In addition, Woodward *et al* ^10^ found there is an impact on self-management of MLTCs for people experiencing socioeconomic deprivation because of the complexities of navigating the health service and finance support systems while experiencing barriers related to their economic, health, and social situations.

Healthcare professionals (HCPs) working in primary care play a key role in supporting self-management; however, the degree to which HCPs view self-management as appropriate and effective differs, which can limit opportunities for shared responsibility and decision making when managing conditions.^11,12^ People experiencing socioeconomic deprivation have reported feeling less able to ask their doctor questions, and believe their perceived socioeconomic situation influences the care they receive and the relationship they have with their healthcare provider.^13,14^ HCPs in deprived areas are more stressed, with higher rates of burnout, as they attempt to care for patients with complex needs in limited time.^15^

HCPs have reported finding the management of MLTCs challenging because of a disorganised and fragmented healthcare system; single-disease guidelines; barriers to shared decision making; and an overall sense of isolation in decision making.^16,17^ General practices serving deprived areas of England receive on average 9.8% less funding per needs-adjusted patient than those in more affluent areas.^18,19^ In addition, there is an unequal distribution of GPs across the UK, with areas of socioeconomic deprivation having lower numbers of GPs.^20^ Researchers in Scotland investigated additional complexities within deprived areas and proposed that whole-system changes are needed to enhance the effectiveness of MLTC support in primary care for people in deprived areas.^21^

Research is needed to understand HCPs’ perspectives of supporting self-management in MLTCs to understand the problem and to find realistic solutions.^21^ The aim of the study was therefore to explore how HCPs in general practices in deprived areas support people to self-manage MLTCs, and the barriers and facilitators of giving best levels of care.

## Method

### Design

The study was guided by an interpretivist paradigm, which posits that reality is socially constructed, with researchers' knowledge and understanding created through subjective interpretations of the data.^22^ In practice, this meant the study goal was not to find a single objective ‘truth’ in the data, but rather to understand the rich, subjective experiences of a range of primary care HCPs from their own perspectives. To explore these varied perspectives, qualitative methodology was employed in the form of semi-structured interviews.^23^

### Patient and public involvement and engagement

Two patient and public involvement and engagement (PPIE) members contributed to all stages of the study by giving feedback on the topic guide, providing important insights surrounding the interpretation of interview data, and development of themes.

### Procedure

Participants were eligible if they worked in GP practices in London and Sheffield, and treated people with MLTCs experiencing socioeconomic deprivation. London and Sheffield were chosen as two diverse areas with pockets of deprivation representing south and north areas of England. For reporting purposes, the Index of Multiple Deprivation categories for each GP practice was looked up by the research team (the first and second authors) on the Fingertips website.^24^ Recruitment was via a study advert shared on social media and circulated through primary care networks and general practices via the research team’s existing contacts. The study advert was also shared with an integrated care board nursing forum and Deep End networks. Recruitment was supplemented by snowball sampling as participants went on to share the study with their own networks.

Eighteen participants were recruited, determined pragmatically based on the time and resources available for the research, but allowing for a breadth of primary care HCP roles and experience to be explored.^25^

A topic guide of open-ended questions and prompts, based on existing literature and research objectives, was developed collaboratively by all authors, including GPs, a general practice nurse, PPIE members, and experienced academic researchers. The topic guide covered the role of the HCP and the wider primary care team in supporting self-management, the challenges and facilitators of supporting people to self-manage, and the additional impact of having multiple conditions and experiencing socioeconomic deprivation. The topic guide was piloted with a GP outside of the research team to check comprehension and relevance of the interview questions.

The researcher (the first author) captured participants’ verbal consent through a JISC Online Surveys consent form, and a Microsoft Word copy of their consent form was emailed to the participant after the interview. Participants gave demographic information (location, place of work, profession, number of years working in primary care, socioeconomic background, and ethnicity) via a JISC Online Survey either before or during the interview. Interviews were conducted by the first and second authors online via Microsoft Teams and were audio-recorded, transcribed, and anonymised.

### Data analysis

Data analysis was underpinned by reflexive thematic analysis, which allowed flexibility for an inductively developed analysis of HCPs’ experiences to be situated within wider socioecological discourses.^26,27^ Analysis occurred in several phases. First, two researchers (the first and second authors) independently analysed interview transcripts, systematically identifying and labelling codes (using NVivo 14^TM^ software) in the data without using pre-existing categories.^26^ The first and second authors met regularly to discuss their coding patterns to develop a more comprehensive and rich understanding of multiple potential interpretations of meaning in the data.^28^ Next, axial coding (borrowed from grounded theory methodology) was conducted by the senior author and the first author to develop codes into a visual map to aid further discussion and begin theme development.^29^ Preliminary themes were shared with all authors for discussion. It was clear from the data that multifaceted individual and environmental factors interact when it comes to self-management of MLTCs. Socioecological theory was proposed by the wider project team as a lens for viewing the data as it provides a framework for understanding how behaviour is not solely determined by individual choices, but is influenced by their relationships, their community context, and broader societal structures.^30^ The first author, second author, and senior author retrospectively applied a socioecological lens to the data by mapping the codes and preliminary themes on the framework. Themes were further refined through discussion with the wider project team.

Reflexivity was used throughout the data collection and analysis process, with the research team (academic researchers, GPs, and a general practice nurse) using written reflections and shared discussions to consider how their own professional and personal experiences and perspectives (for example, the first author as a trustee of a charity in a deprived area) may have an impact on the design, conduct, analysis, and write-up of the study.^27^

## Results

Eighteen primary care HCPs from 17 different practices took part in interviews lasting between 30 and 53 min. Participant demographics are presented in Table 1.

**Table 1.** Participant demographics (*N* = 18)^a^

**Category**	**Values**
**Recruitment area**	
Greater London	8
Sheffield	10
**Profession**	
GP	7
GP registrar	1
Advanced nurse practitioner	2
Clinical pharmacist	2
General practice nurse	3
Occupational therapist	1
First-contact physiotherapist	2
**Years working in primary care, years: median (range)**	10.2 (1–43)
**General practice deprivation level** ^24^	
Most deprived decile^b^	8
Second most deprived decile	4
Third most deprived decile	2
Fourth most deprived decile	3
Sixth most deprived decile	1
**Socioeconomic background^c^**	
Lower socioeconomic background	2
Intermediate background	1
Professional background	15
**Ethnicity**	
White, English, Welsh, Scottish, Northern Irish or British	9
Any other White background	3
Indian	3
Mixed or multiple ethnic background	2
Bangladeshi	1

^a^Data are *n* unless otherwise specified. ^b^Deciles are calculated by ranking the 32 844 neighbourhoods in England from most deprived to least deprived and dividing them into 10 equal groups. One participant worked in a GP practice in a fourth-least-deprived decile area, but described how their practice served people seeking asylum, whom they perceived to be experiencing socioeconomic deprivation. ^c^What was the occupation of the main/highest income earner in the household when you were 14 years?

Four themes were developed (Figure 1) and quotations are given to illustrate narrative, with data extracts identified by participant number, HCP role, and years of experience working in primary care reported for context.

**Figure 1.**
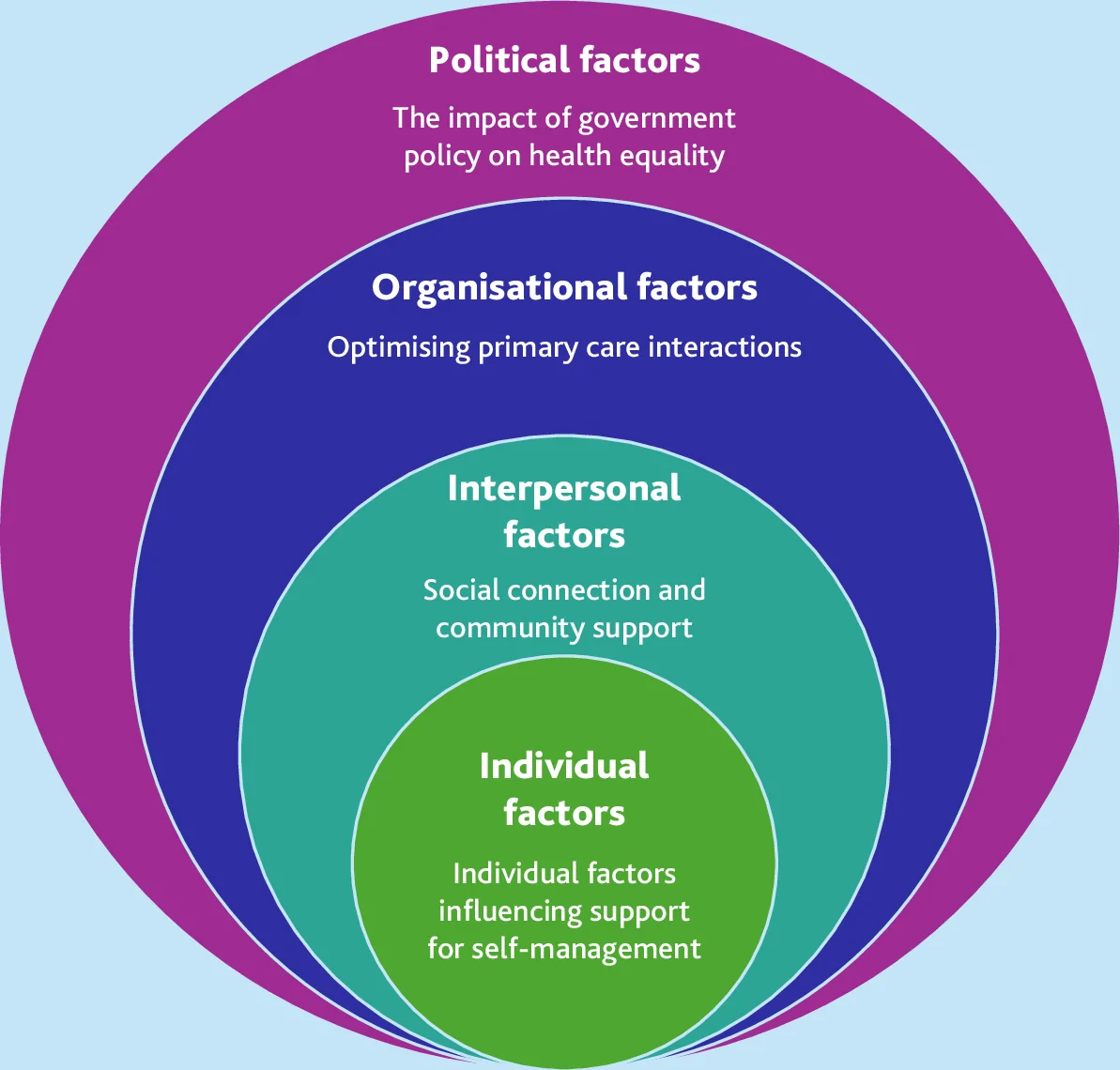
A socioecological model of factors affecting healthcare professional support for self-management of multiple long-term conditions.

### Theme one: individual factors influencing support for self-management

Individual and cultural differences in health beliefs and health-related behaviours were described as having an impact on the development and management of some long-term conditions and being difficult to navigate in primary care consultations. For example, avoiding seeking medical care was seen as a social norm among some socioeconomically deprived groups, indicating HCPs felt concerned that these individuals were not accessing the self-management support they need:

*‘… white older men who avoid the doctors because you go to the doctors, you’ll find out you’ve got lung cancer and then you’ll die a year later.’* (P16, GP, 6 years’ experience in primary care)

When individuals with MLTCs did engage in primary care services, the majority of HCPs described their role in providing information to support individuals to make informed decisions about managing their conditions. A few HCPs mentioned lack of guidelines on how diseases interact with each other:

*‘There’s a real lack of evidence on managing conditions simultaneously. There’s great evidence on how to manage heart failure and diabetes or COPD* [chronic obstructive pulmonary disease] *… but when you put them all together there’s not good evidence about how to manage them in combination.’* (P09, GP, 4 years’ experience in primary care)

Many also highlighted that added complexities of socioeconomic deprivation had an impact on their ability to provide accessible information to the same level as in more affluent communities:

*‘We’ve got lots of trusted resources, which in non-deprived areas work quite well, just sending* [them] *out nice and quickly. But the language barriers, technology barriers, are a massive issue.’* (P07, first-contact physiotherapist, 7 years’ experience in primary care)

Many HCPs shared that digital barriers, such as limited internet or smartphone access and limited knowledge of using digital self-management devices, have an impact on individuals’ ability to engage in self-management. HCPs noted that support with digital skills and access was needed for some of their patients in this population:

*‘I know we’re moving towards digital health care … but people find that very difficult to engage with, especially when people often don’t have access to smartphones or internet in this area.’* (P14, clinical pharmacist, 5 years’ experience in primary care)

One HCP described work being done in their area to provide information in an accessible way:

*‘We’re trying to have an understanding of the way the community like to source health information … social media sites are used quite widely, so we’re trying to use those things.’* (P09, GP, 4 years’ experience in primary care)

Alongside giving medical information to help individuals understand their conditions, all HCPs spoke about giving lifestyle and behavioural advice to help manage these conditions long term. However, some pointed out that, even when individuals have the information and skills needed, their socioeconomic circumstances can affect implementation of self-management. Poor access to green spaces, living in unsafe neighbourhoods, having low income, and limited access to resources were frequently discussed by HCPs as barriers to managing health conditions:

*‘... a large proportion of people already know what a healthy diet looks like, they know what exercise is, but you go to the store … all of the cheap food is junk food … you have got to inhale exhaust fumes across multiple busy roads to get to the nearest area of green space, you can’t afford a gym membership … it’s people being set up to fail. Doesn’t matter how much advice you give them.’* (P17, GP, 10 years’ experience in primary care)

Although behaviour-change conversations were deemed challenging, especially in time-limited appointments, HCPs suggested they are ‘empowering’ for patients and found them ‘rewarding*’*. However, it was highlighted that behaviour-change conversations need to consider people’s socioeconomic circumstances:

*‘It’s very easy to turn people off … people feel that we don’t really understand the way they live.’* (P04, GP, 43 years’ experience in primary care)

### Theme two: social connections and community support

Encouraging individuals to draw on their own support network was viewed by HCPs as important for the medical, emotional, and behaviour-change elements of self-management of MLTCs, especially when socioeconomic deprivation led to additional stresses and strains on everyday life:

*‘It’s not self-management, it’s community management … you need support. Whenever you have an engaged family member or friend … to help them remember what you’ve said and then encourage them to actually do it, it can be really powerful.’* (P11, GP, 6 years’ experience in primary care)

HCPs expressed admiration for unpaid carers, especially when they were also experiencing lack of access to or control over finances, resources, opportunities, or support. Coordinating appointments with family members or carers was seen as a facilitator to supporting self-management, particularly in cases where patients were less able to express themselves and their own needs. However, some HCPs pointed out carers sometimes had different priorities from the individual, may interpret symptoms differently, and in some cases could undermine the work of the HCP:

*‘It can change the dynamic … because I’ve tried to get them to elicit the responses … if you’ve got a carer there that’s telling you “you should change”, you can get more resistant to the change.’* (P03, advanced nurse practitioner, 5 years’ experience in primary care)

HCPs pointed out that levels of existing care and support varied for their patients and occasionally healthcare workers may be the most supportive relationships the patients have experienced:

*‘We do see a lot of people who haven’t really felt cared for by anybody in their life. We’ve got a lot of people who had really complex childhoods and complex relationships … sometimes some of the most consistent caring relationships they’ve had are with our practice nurse.’* (P16, GP, 6 years’ experience in primary care)

All HCPs felt unable to provide enough holistic support for individuals with their MLTC management within the remit of their professional role, and investment into community-based health education and social support was highlighted as a key facilitator for prevention and management of MLTCs. HCPs discussed the importance of shifting care out of the surgery into the community:

*‘There’s a chronic pain worker who will get people to the chronic pain café … It’s not just a problem that can be solved by a doctor, so let’s take it out of the GP surgery and re-empower people … there’s been real success with that.’* (P09, GP, 4 years’ experience in primary care)

Culture-specific groups and peer-led groups were mentioned as important sources of trusted information and support, and a way to help individuals to self-manage their conditions:

*‘Sometimes having a Bangladeshi diabetes group is more useful than anything because of their specific beliefs, or that they have more trust in people from their own community.’* (P02, GP registrar, 2 years’ experience in primary care)

However, community-based support groups and services were not available in all areas, and were often short term and underfunded, making it difficult for staff and patients to keep up with and navigate:

*‘If we really care about these people, we’d be investing in these organisations, because without them and the support that they give the patients, there’s absolutely no way that we could make inroads into their health.’* (P04, GP, 43 years’ experience in primary care)

Many HCPs mentioned the role of social prescribers, link workers, or community connectors, who were used quite differently across practices depending on levels of affluence:

*‘They’ve got far more resources, they didn’t really know what to do with their social prescribers, you know taking them on a little rambling walk … but in* [more deprived location]*, all our one was doing was food banks and housing crisis and immigration stuff.’* (P01, GP, 19 years’ experience in primary care)

One HCP described social prescribers as a ‘bridge’ linking individuals to community-based support, but pointed out that sometimes there might not be the right support in the community to link them to. Two HCPs highlighted that social prescribers were becoming overwhelmed by demand for support:

*‘We used to want to refer to the social prescribers, they’d get seen quite quickly and then signposted on to things, but there’s quite a bit of a delay now. I think they’re just overwhelmed.’* (P16, GP, 6 years’ experience in primary care)

### Theme three: optimising primary care interactions

Organisational factors related to the structure, policies, and practices of primary care were prevalent in HCPs’ interviews, with a focus on how these factors had an impact on the HCPs’ ability to support MLTC self-management for individuals experiencing socioeconomic deprivation. All HCPs spoke about the limitations of primary care appointments (for example, length, frequency, and accessibility), highlighting the barriers that socioeconomic deprivation often added, which required more time and resources:

*‘If they didn’t have any of those barriers, you can get from nought to ten in fifteen minutes … but with all those barriers, it feels like you can only get to about nought to one … that can be frustrating for us, and sad, but more importantly it means that they don’t get the care that they need or that we’re trying to give.’* (P01, GP, 19 years’ experience in primary care)

Some practices were trying to offer longer or more frequent appointment times for individuals experiencing MLTCs and socioeconomic deprivation, which HCPs cited as ‘acknowledgement that our patients are complex’ and ‘they need a bit of extra time and care’*.* Other adaptations included offering Saturday clinics, recruiting staff whose role is to help patients navigate appointments, supporting with travel costs, and making home visits. One pharmacist spoke about the distinct role of an outreach team of clinicians developed specifically to meet the complex needs of their local population:

*‘… going to people’s houses, seeing how they live … feeding back to us: what does this patient need? Helping us engage … bringing them into practice, getting them into the appointments.’* (P14, clinical pharmacist, 5 years’ experience in primary care)

However, some commented that clinical targets (such as NHS England’s Quality and Outcomes Framework) were often at odds with providing person-centred care and behaviour-change support:

*‘I would say government targets are extremely difficult and put pressure on us to medicalise everybody very quickly without giving people time to manage something with lifestyle factors.’* (P06, general practice nurse, 12 years’ experience in primary care)

Having time to listen to individuals and understand more about their lives was seen as important for supporting them with MLTC management, and many HCPs spoke about continuity of care being ‘paramount’, ‘crucial’, and a facilitator to build trust with individuals experiencing socioeconomic deprivation:

*‘Continuity is important because if you haven’t seen a patient multiple times, you often won’t know the intricacies of their circumstances and you won’t know about the challenges they faced.’* (P17, GP, 10 years’ experience in primary care)

Developing trusted relationships was seen to facilitate health-seeking behaviours. However, building relationships with individuals from more deprived and ethnically diverse communities was seen by some HCPs to be challenging:

*‘I’ve worked in areas that are a lot more affluent, and people generally feel like society and the system is working for them — that people who are in positions of responsibility have their best interests at heart. Working with more deprived communities, it takes a longer time to produce that trust.’* (P17, GP, 10 years’ experience in primary care)

It was clear that many HCPs found working in deprived communities hard, and some spoke about the complex medical and socioeconomic needs of the individuals they support contributing to high staff turnover, understaffing, and burnout:

*‘People really struggle, and don’t want to commit long term to working in areas like this. It’s often something they’ll do for a few years and then move on somewhere a bit easier, because you can’t really cover up the fact that it’s just incredibly hard work.’* (P04, GP, 43 years’ experience in primary care)

HCPs spoke of the support they received through staff networks, training, mentoring, and team debriefing as being a source of strength in a difficult working environment:

*‘I think there’s a certain type of practitioner that tends to want to work in those areas. The ones that I know tend to be very committed and inspirational, and so that really brings everyone together into a network of support as well.’* (P01, GP, 19 years’ experience in primary care)

### Theme four: the impact of government policy on health equality

Nearly all HCPs discussed how political and policy factors influence the social drivers that have an impact on the development and management of MLTCs in areas of socioeconomic deprivation (such as poor environmental conditions, unsafe neighbourhoods, and lack of healthy food options). Most also spoke about how political and macroeconomic decisions have an impact on their ability to support individuals to self-manage their conditions. HCPs’ frustrations reflected a systemic issue of underfunding of primary care and indicated that the formula used to calculate funding distribution across practices (the Carr-Hill index) is perceived as unfair and outdated:

*‘That’s where a lot of the GPs are very angry about the inequality. Some of the payments, the Carr-Hill index hasn’t been revised for so long and it actually disadvantages some of the surgeries.’* (P01, GP, 19 years’ experience in primary care)

Half of the HCPs expressed their concern that poor housing (such as unsafe, overcrowding, and mould) was an issue linked closely to the development of MLTCs, whereas adequate housing was seen as important for good health and for MLTC prevention and management:

*‘I think housing is imperative for physical health … I have patients who live in overcrowded homes, with heating that’s on bare minimum.’* (P13, first-contact physiotherapist, 4 years’ experience in primary care)

Housing needs were also mentioned by HCPs to highlight the different priorities individuals and practitioners often have during a consultation. A lack of adequate housing for this population is indicative of structural inequalities that HCPs are having to navigate when working to support individuals. HCPs described a common experience of writing letters of concern to patients’ housing associations about the level of mould in their properties, but this was time consuming, and some had been told their letters would not be accepted:

*‘It doesn’t make any difference, it takes a lot of doctors’ time writing letters and I know that it doesn’t make a difference, which is hard.’* (P03, advanced nurse practitioner, 5 years’ experience in primary care)

Many HCPs emphasised that, with levels of inequality worsening, supporting those with MLTCs living in areas of socioeconomic deprivation is becoming even harder:

*‘I see things changing and getting worse. Those that were struggling before are really struggling. The ones that weren’t struggling at all are now struggling. It’s becoming more and more challenging because you’ve only got so many resources — and so many people need it.’* (P12, advanced nurse practitioner, 9 years’ experience in primary care)

It was clear that many HCPs felt that a more preventive public health model is urgently needed to decrease development of MLTCs, including early intervention, public health messaging, and adequate social welfare:

*‘Prevention, education … by the time people already have MLTCs you’re already in damage limitation mode … ensuring kids get a really good start to life and have early years education, ensuring that families with children have enough money for adequate clothing, housing, and food … in the longer term, those things will pay off more.’* (P17, GP, 10 years’ experience in primary care)

## Discussion

### Summary

This paper aimed to explore primary care HCPs’ perspectives and experiences of management of MLTCs in people experiencing socioeconomic deprivation. Individual factors such as attitudes and values affected the individual’s access to, and implementation of, self-management advice, which in turn had an impact on the HCP’s support for self-management. Providing continuity of care and building trust through offering socioeconomically sensitive and non-judgemental self-management education and support was seen as essential. Social connection and community-based support was seen by HCPs as important for self-management but was not always available or accessible. HCPs described adaptations made at clinician or practice level to provide the best level of care to their communities, such as longer or more frequent appointments, patient navigators, and outreach teams. HCPs also discussed the impact on their own wellbeing of supporting patients with complex needs in an inequitably funded healthcare service. Underfunding of primary care in deprived areas was seen to be limiting the level of MLTC self-management support that HCPs could provide to address the complexity of individuals’ needs, with reference to longer and more frequent consultations to allow for person-centred care and behaviour-change conversations.

### Strengths and limitations

Recruiting through well-established existing networks and snowball sampling led to a sample diverse in experience and ethnicity, with a near-even split between Sheffield and London. Further research could explore the impact on self-management support where socioeconomic deprivation exists in rural and coastal areas. The current study includes a range of HCP perspectives, representing the varied primary care teams who support self-management of MLTCs, but recruiting via existing networks may have led to participation from those who are already engaged in research and passionate about challenging health inequalities. Having a wide research team and including PPIE members throughout the study, and drawing on elements of reflexive thematic analysis and grounded-theory methodology, allowed for a rich consideration of the data, combining a fresh perspective with existing theory and evidence.

### Comparison with existing literature

In line with recent research, this study provides support that the psychosocial needs of individuals experiencing socioeconomic deprivation often dominate primary care interactions.^31^ Existing research has shown that HCPs require education, training, and resources to improve their cultural competence and feel more confident in supporting individuals to develop self-management skills.^32^ Culturally sensitive communication has been found to aid in establishing and maintaining a trusting therapeutic relationship^33^ and existing research shows that trust, particularly across cultural and societal divides, and continuity of care are key factors in providing quality care for individuals in areas of socioeconomic deprivation.^34,35^ The current study adds support to these findings from the perspective of HCPs supporting MLTC self-management.

Research has found that more time for consultations with patients with complex problems can reduce GP stress and enable HCPs to offer more preventive and coordinated care, which is in line with the findings of the current study.^21^ Having staff employed to help individuals navigate health and social care systems was also seen as a facilitator to supporting MLTC self-management, a finding that supports recent literature where these roles have been found to foster trust and empowerment within underserved communities, improving healthcare access and quality through advocacy, care coordination, and addressing disparities associated with language barriers and cultural differences.^36,37^

The current study showed that having up-to-date MLTC guidance that can be shared with individuals using a variety of different mediums and methods is required when supporting self-management but is not always available or not easily accessible because of digital exclusion, health literacy, and language or cultural barriers. Previous research indicates individuals experiencing MLTCs and socioeconomic deprivation identify these same issues as barriers to self-management.^8,10^ Recent research suggests that 2.8 million people in the UK do not have access to the internet, and that 8% of internet users lack confidence online.^38,39^ Studies indicate that socioeconomic disparities exacerbate digital inequalities, which in turn can further worsen existing health and social inequalities.^40^ In light of the UK government’s proposed long-term health plan^41^ to shift from analogue to digital, the current study findings provide important support to bolster recent evidence that digital technologies designed to improve health can reinforce inequalities within underserved populations if implementation does not consider the structural, economic, social, and behavioural factors contributing to digital exclusion.^42,43^

In line with the UK government’s proposed long-term health plan^41^ to move care closer to communities, HCPs described delivering and signposting to preventive health care and self-management education and support in community settings. Although our results showed many HCPs thought being able to refer patients to community-based education and support was important for supporting MLTC self-management, other literature suggests that HCPs may be reluctant to do so, citing concern about fragmentation of care and doubting the benefits for patients.^11,44^ However, recent systematic reviews have shown that interventions specifically tailored to socioeconomically deprived groups report significant improvements in clinical outcomes.^45,46^ Despite its best efforts, the healthcare system is unable to address the root causes of health disparities without significant equality-focused policy change and equitable resourcing (such as reforming funding formulas and financial incentive systems).^18,47^ Syndemic intervention is needed to strengthen efforts towards prevention and self-management of MLTCs by addressing both inequality and the treatment of disease symptoms.^48^

### Implications for research and practice

This study highlights the need to prioritise socioeconomically sensitive approaches in the support of people with MLTCs through continuity, trust, and person-centred care in primary care settings, particularly in deprived areas. Future research should focus on evaluating interventions or approaches that allow flexibility in consultations (such as longer appointments), enhance health system navigation, and integrate community-based support. Ensuring inclusivity around digital skills, language, and health literacy is essential when developing self-management resources. Policymakers must address structural inequalities, particularly the underfunding of primary care in deprived areas, which limits the capacity of HCPs to respond to complex patient needs. Investment in workforce wellbeing, culturally and socioeconomically appropriate education, and navigation roles are vital. Ultimately, syndemic-informed strategies that consider social determinants are needed to reduce the cyclical relationship of socioeconomic inequality and improve outcomes in MLTCs.

In conclusion, the findings of this study highlight how primary care HCPs working in socioeconomically deprived areas of two English cities are navigating the discrepancy between what is needed for providing support for MLTC self-management and what is possible within current systems. A socioecological approach highlights that, despite best efforts, individuals and the healthcare system supporting them are unable to address the root causes of MLTC health disparities without significant policy change and equitable resourcing.
